# A Phase 2 Clinical Trial of Trametinib and Low-Dose Dabrafenib in Patients with Advanced Pretreated *NRAS^Q61R/K/L^* Mutant Melanoma (TraMel-WT)

**DOI:** 10.3390/cancers13092010

**Published:** 2021-04-22

**Authors:** Gil Awada, Julia Katharina Schwarze, Jens Tijtgat, Giuseppe Fasolino, Hendrik Everaert, Bart Neyns

**Affiliations:** 1 Department of Medical Oncology, Vrije Universiteit Brussel (VUB), Universitair Ziekenhuis Brussel (UZ Brussel), 1090 Brussels, Belgium; gil.awada@uzbrussel.be (G.A.); juliakatharina.schwarze@uzbrussel.be (J.K.S.); jens.tijtgat@uzbrussel.be (J.T.); 2 Department of Ophthalmology, Vrije Universiteit Brussel (VUB), Universitair Ziekenhuis Brussel (UZ Brussel), 1090 Brussels, Belgium; giuseppe.fasolino@uzbrussel.be; 3 Department of Nuclear Medicine, Vrije Universiteit Brussel (VUB), Universitair Ziekenhuis Brussel (UZ Brussel), 1090 Brussels, Belgium; hendrik.everaert@uzbrussel.be

**Keywords:** advanced melanoma, *NRAS* mutation, trametinib, dabrafenib, skin toxicity, phase 2 clinical trial

## Abstract

**Simple Summary:**

MEK-inhibitor monotherapy has activity in advanced *NRAS^Q61R/K/L^* mutant melanoma but is associated with dose-limiting cutaneous toxicity. The combination of a BRAF- with a MEK-inhibitor at their full dose (as in *BRAF^V600E/K^* mutant melanoma) has low cutaneous toxicity. We hypothesized that a low dose of the BRAF-inhibitor dabrafenib can mitigate the skin toxicity associated with a full dose of the MEK-inhibitor trametinib in patients with advanced *NRAS^Q61R/K/L^* mutant melanoma who progressed after treatment with immune checkpoint inhibitors. The results of this two-stage phase 2 trial show that addition of a low dose of dabrafenib effectively mitigates the skin toxicity associated with trametinib. This combination is, however, insufficiently active in patients with advanced *NRAS^Q61R/K/L^* mutant melanoma. The combination of low-dose dabrafenib plus full-dose trametinib can be of further interest for the treatment of MEK-inhibitor-sensitive tumors.

**Abstract:**

Background: MEK-inhibitor monotherapy has activity in advanced *NRAS^Q61R/K/L^* mutant melanoma but is associated with dose-limiting cutaneous toxicity. The combination of a BRAF- with a MEK-inhibitor at their full dose (as in *BRAF^V600E/K^* mutant melanoma) has low cutaneous toxicity. It is unknown whether a low dose of BRAF-inhibitor can mitigate the skin toxicity associated with full-dose MEK-inhibitor treatment in patients with advanced *NRAS^Q61R/K/L^* mutant melanoma. Methods: This two-stage phase 2 clinical trial investigated trametinib 2 mg once daily in patients with advanced *NRAS^Q61R/K/L^* mutant melanoma who were pretreated with immune checkpoint inhibitors. In case of trametinib-related cutaneous toxicity, low-dose dabrafenib (50 mg twice daily) was added to prevent recurrent cutaneous toxicity (pre-amendment). Following an amendment, trametinib was combined upfront with low-dose dabrafenib (post-amendment). Objective response rate (ORR) served as the primary endpoint. Results: All 6 patients enrolled pre-amendment developed trametinib-related cutaneous toxicity, necessitating treatment interruption. Combining trametinib with low-dose dabrafenib prevented recurrent skin toxicity thereafter. Trametinib-related skin toxicity was effectively mitigated in all 10 patients post-amendment. In all 16 included patients, the ORR and disease control rate was 6.3% (1 partial response) and 50.0%, respectively. The trial was halted after the first stage. Conclusions: Combining full-dose trametinib with low-dose dabrafenib can mitigate MEK-inhibitor-related skin toxicity but was insufficiently active in this patient population. This combination can be of further interest for the treatment of MEK-inhibitor-sensitive tumors.

## 1. Introduction

No therapy has shown to increase overall survival (OS) of patients with advanced *BRAF^V600^* wild-type melanoma (50% of melanoma patients) who progress beyond treatment with programmed cell death 1 (PD-1) and cytotoxic T-lymphocyte-associated antigen 4 (CTLA-4) immune checkpoint inhibitors (ICI).

*NRAS^Q61R/K/L^* mutations, present in approximately half of patients with advanced *BRAF^V600^* wild-type melanoma, are mutually exclusive with *BRAF^V600^* mutations and activate the mitogen-activated protein kinase pathway (MAPK- or RAS-RAF-MEK-ERK-pathway) through canonical activation of RAF, MEK and ERK [1,2,3]. Phosphorylated ERK will subsequently activate multiple processes involved in cellular proliferation, motility and metastasis [4]. In the randomized phase 3 NEMO trial, comparing the MEK-inhibitor binimetinib to dacarbazine chemotherapy in patients with advanced *NRAS^Q61R/K/L^* mutant melanoma, binimetinib treatment resulted in a significantly improved objective response rate (ORR, 15% versus 7%) and median progression-free survival (PFS, 2.8 versus 1.5 months, *p* < 0.001), but not OS (11.0 versus 10.1 months, *p* < 0.50) [5]. In a subgroup analysis, immunotherapy-pretreated patients had a better PFS with binimetinib (hazard ratio [HR] 0.46 [95% confidence interval [95% CI] 0.26–0.81]). Preclinical data show that adding a BRAF- to a MEK-inhibitor inhibits ERK-phosphorylation and cell growth and induces apoptosis in *NRAS* mutant melanoma models by inducing endoplasmic reticulum stress [6].

Treatment with MEK-inhibitors is associated with a distinct toxicity profile, most commonly cutaneous adverse events (acneiform dermatitis, skin fissures, paronychia), but also including fatigue, muscular, cardiovascular, ocular and digestive toxicity. In the NEMO trial, adverse events resulted in dose reductions in 61%, dose interruptions in 58% and treatment discontinuations in 20% of patients [5]. MEK-inhibitor-related rash was diagnosed in 65% of cases (grade 3–4 8%) and led to dose interruptions and dose reductions. In phase 3 trials in advanced *BRAF^V600E/K^* mutant melanoma, the incidence of rash is substantially lower with the combination of the BRAF-inhibitor dabrafenib and the MEK-inhibitor trametinib (all-grade 28%) as compared to trametinib monotherapy (all-grade 57%), at the same trametinib dosing [7,8].

The phase 2 TraMel-WT trial investigates the efficacy and safety of trametinib in patients with advanced *BRAF^V600^* wild-type, *NRAS^Q61R/K/L^* mutant or wild-type melanoma who previously failed ICI treatment. In order to mitigate trametinib-related skin toxicity, a low dose of dabrafenib is added to trametinib. We hypothesize that the addition of a low dose of dabrafenib will lead to better tolerance of, and therefore a potentially higher exposure to, trametinib. This article reports the results of patients included in the *NRAS^Q61R/K/L^* mutant cohort.

## 2. Methods

### 2.1. Study Design and Patient Population

This single-center, two-stage, dual-stratum, open-label phase 2 clinical trial (NCT04059224) was conducted at the Universitair Ziekenhuis Brussel (Brussels, Belgium) and included adult patients with advanced (unresectable or metastatic) *BRAF^V600^* wild-type, *NRAS^Q61R/K/L^* mutant melanoma who had confirmed progressive disease (PD) following or who were ineligible for treatment with PD-1 and/or CTLA-4 ICI (for example, due to severe immune-related adverse events).

Eligible patients must have had an Eastern Cooperative Oncology Group Performance Status (ECOG PS) of 0–2; adequate baseline organ function and presence of archival or newly obtained melanoma tissue for confirmatory mutational testing. Major exclusion criteria were patients with uveal melanoma, prior treatment with MAPK-pathway inhibitors, the presence of clinically active brain metastases, and presence of uncontrolled cardiovascular and/or ocular diseases. All inclusion and exclusion criteria are summarized in Table S1.

### 2.2. Procedures and Study Treatment

All patients were screened for eligibility by clinical examination, blood analysis, electrocardiography (ECG), transthoracic echocardiography (TTE), ophthalmic examination (including optical coherence tomography), whole-body 18-fluorodeoxyglucose positron emission tomography/computed tomography (^18^F-FDG-PET/CT) and magnetic resonance imaging of the brain in case of brain metastases. A plasma sample was obtained for circulating tumor DNA (ctDNA) analysis (see below). The *NRAS^Q61R/K/L^*/*BRAF^V600^* mutational status was confirmed on tumor tissue using the Idylla™ NRAS-BRAF Mutation Test (Biocartis, Mechelen, Belgium) or genomic sequencing by local institutional standards.

All patients were treated with trametinib 2 mg once daily orally. Dabrafenib 50 mg twice daily orally (“low-dose”) was added to trametinib in case of dose-limiting trametinib-related cutaneous toxicity. Dabrafenib dosing could be increased in case of insufficient control of trametinib-related cutaneous toxicity to 100 mg or 150 mg twice daily. In June 2019, the trial was amended to administer dabrafenib 50 mg twice daily upfront with trametinib. Patients enrolled before this amendment were post-hoc analyzed as an exploratory cohort, while patients enrolled after this amendment were considered as the population on which the primary endpoint of efficacy was tested.

Patients were evaluated on a regular basis with a clinical examination, blood analysis, ECG, TTE and ophthalmic examination. Every 8 weeks, response assessments were performed. Study therapy was continued until confirmed PD, unacceptable toxicity or withdrawal of consent. The database was locked on 28 October 2020.

### 2.3. Endpoints

The primary endpoint is the confirmed ORR, per Response Evaluation Criteria In Solid Tumors version 1.1 (RECIST v1.1) [9]. Secondary endpoints are to estimate the PFS and OS, and to characterize the incidence and severity of adverse events (AE, graded by the Common Terminology Criteria for Adverse Events [CTCAE] version 4.03) of trametinib and dabrafenib, with special interest in the possibility to manage trametinib-related treatment-limiting skin toxicity by adding dabrafenib. Trametinib and dabrafenib daily dose were calculated as the amount of drug given per day (mg) that patients were on study treatment (including treatment interruptions); the dose intensity (%) reflects the ratio between the administered and the hypothetical full-dose intensity in the patient population.

Exploratory endpoints include the predictive/prognostic role of *NRAS^Q61R/K/L^* ctDNA and the association between whole-body ^18^F-FDG-PET/CT-assessed total metabolic tumor volume (TMTV) at baseline and treatment outcome.

### 2.4. ctDNA Analysis

The method of analysis of baseline plasma *NRAS^Q61R/K/L^* mutant ctDNA has been described in a previous article by our group and was dichotomized as detectable or undetectable [10].

### 2.5. Total Metabolic Tumor Volume Analysis

In patients who had undergone baseline whole-body ^18^F-FDG-PET/CT, the TMTV was calculated as the sum of all tumor-associated voxels with a standardized uptake value (SUV) above the mean SUV measured in a reference region in normal liver tissue plus 3 standard deviations of tumor lesions sized ≥1 mL (Syngo.via version VB40, Siemens Healthineers GmbH, Erlangen, Germany).

### 2.6. Statistical Analysis

The sample size was calculated according to a Simon’s two-stage optimal design. The null hypothesis that the true ORR is 10% will be tested against a one-sided alternative that the minimal ORR on the experimental therapy is 30%. In the first stage, 10 patients will be accrued. If there is ≤1 confirmed response, the study will be stopped for futility. Otherwise, 19 additional patients will be accrued for a total of 29 patients in the second stage. The null hypothesis will be rejected if >5 responses are observed in these 29 patients. This design yields a type I error rate of 0.05 and a power of 0.80.

This predefined statistical analysis of the primary endpoint (ORR) was eventually applied to patients enrolled after the amendment (trametinib and low-dose dabrafenib combined upfront). Patients who were enrolled before the amendment were considered as part of an exploratory analysis on the intent-to-treat population. PFS and OS were estimated using the Kaplan–Meier method (SPSS Statistics version 27, IBM, Armonk, NY, USA).

## 3. Results

### 3.1. Baseline Characteristics

**Figure 1:**
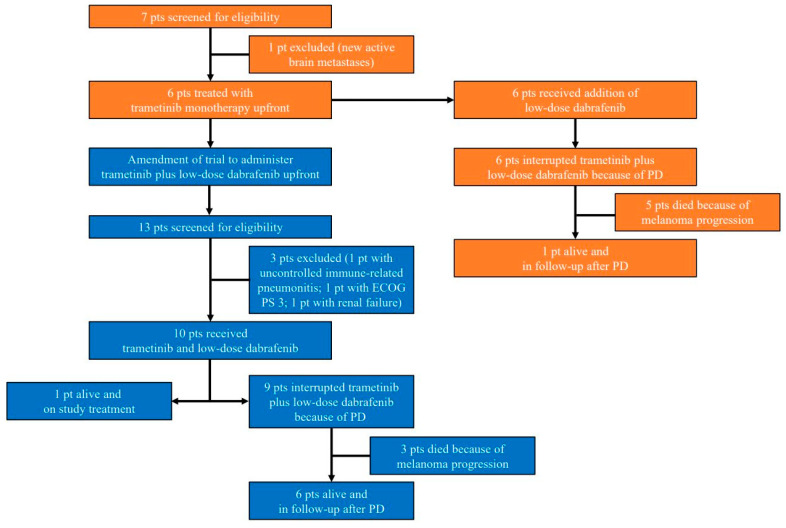
CONSORT-diagram. Patients enrolled prior to the trial amendment are depicted in orange, patients enrolled after the trial amendment and who were part of the first stage of the study are depicted in blue. Abbreviations: ECOG PS: Eastern Cooperative Oncology Group Performance Status; PD: progressive disease; pt(s): patient(s).

Between January 2019 and August 2020, 16 patients initiated study treatment: 6 patients were enrolled prior to the amendment (receiving trametinib upfront with add-on of low-dose dabrafenib in case of trametinib-related cutaneous toxicity). Subsequently, 10 additional patients were enrolled after the trial amendment (receiving trametinib and low-dose dabrafenib upfront) and were considered for addressing the primary endpoint of efficacy (Figure 1). The baseline characteristics are summarized in Table 1.

### 3.2. Treatment Disposition

**Figure 2:**
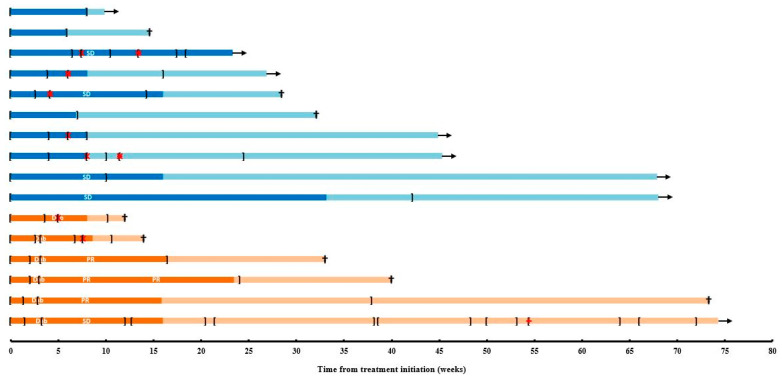
Swimmer plot depicting progression-free (dark orange) and overall survival (light orange) from treatment initiation in the first 6 patients enrolled prior to trial amendment and progression-free (dark blue) and overall survival (light blue) in the 10 patients enrolled after the trial amendment. Intervals on and off study therapy are depicted with square brackets ([…]). Black arrow: alive; black cross: death; Dab: addition of low-dose dabrafenib in the first six patients; PR: partial response; red X: dose reduction trametinib; red +: dose reduction dabrafenib; SD: stable disease.

All enrolled patients (*n* = 16) initiated study treatment according to the protocol (Figure 1). Among the first 6 patients (initiating trametinib as a monotherapy), and subsequent 10 patients (initiating trametinib together with low-dose dabrafenib), respectively 6 (100.0%) and 6 (60.0%) patients were in need of interrupting trametinib treatment and respectively 2 (33.3%) and 5 (50.0%) patients required a trametinib dose reduction because of treatment-related AE (Figure 2). Two (20.0%) patients who initiated trametinib after the trial amendment required a second dose reduction of trametinib. Among the first 6, and subsequent 10 patients, the administered median daily dose and dose intensity of trametinib were 1.8 mg (range 1.5–1.9) and 90%, and 1.4 mg (range 1.0–2.0) and 75%, respectively.

All 6 patients who initiated trametinib as a monotherapy added on low-dose dabrafenib at the time of treatment-limiting skin toxicity (after a median of 3.1 weeks [range 2.9–5.0] of trametinib monotherapy) (Figure 2). Two patients were in need of a dabrafenib treatment interruption (dabrafenib was always interrupted together with trametinib), one patient required a dabrafenib dose reduction. The median daily dose of dabrafenib was 100 mg (range 85.2–100.0; 93% dose intensity). Among the subsequent 10 patients who initiated trametinib and low-dose dabrafenib together, the median daily dose of dabrafenib was 68.8 mg (range 47.8–100.0; 73% dose intensity). Four out of ten patients required a dabrafenib dose reduction.

For the first 6 and subsequent 10 patients, the median duration of trametinib treatment was 20.2 weeks (range 10.1–72.0) and 12.0 weeks (range 5.9–42.1), respectively. The median duration of low-dose dabrafenib treatment was 17.1 weeks (range 5.1–68.7) and 12.0 weeks (range 5.9–42.1), respectively.

Seven patients (4 out of the first 6 and 3 out of the subsequent 10 patients) were treated beyond first progression (respectively for a median of 12.1 weeks [range 2.0–56.0], and 9.0 weeks [range 8.0–16.4 weeks]). Two of these patients were treated concurrently with radiation therapy for oligoclonal patterns of progression. At the time of database lock, 15 patients had stopped study treatment because of PD, none for reasons of toxicity, and one patient who was treated with trametinib and low-dose dabrafenib upfront was continuing study treatment.

### 3.3. Safety

All patients experienced AE (Table 2). Grade 3–4 AE were observed in 50.0% of patients treated prior to the amendment, and in 40.0% of patients enrolled after the amendment. There were no grade 5 AE.

All of the first 6 patients developed a trametinib-related acneiform rash (5 grade 2; 1 grade 1). Although these were not considered to be serious adverse events nor to be high-grade (grade 3–5 by CTCAE), they were sufficiently symptomatic, disfiguring and interfering with the quality of life to necessitate trametinib interruption and supportive care (oral minocycline, topical metronidazole). Following recovery to grade 0–1, all patients reinitiated trametinib at the same dose with low-dose dabrafenib (50 mg twice a day). Two patients developed a relapse of acneiform rash (grade 1) that did not necessitate treatment interruption, and were treated with topical therapy only. No dose escalations of dabrafenib to control for trametinib-related skin toxicity were necessary. In the subsequent 10 patients, limited acneiform rash was observed in 3 patients (33.3%, all grade 1) which was managed with topical therapy only. Other AE that were observed were fatigue and asymptomatic creatine phosphokinase increase (both 66.7%), and lipase increase, anemia and fever (all 50.0%) in the first 6 patients, and asymptomatic creatine phosphokinase increase (60.0%), and fatigue and diarrhea (both 40.0%) in the subsequent 10 patients. AE of special interest include one patient with acute kidney injury caused by histologically confirmed acute tubulointerstitial nephritis. A recovery of the renal function was observed following the initiation of corticosteroid treatment. An additional patient developed a drug-related pneumonitis that also responded to corticosteroids.

In the first six patients, two patients required a dose reduction of trametinib (reversible central serous retinopathy, and decreased ejection fraction). One patient needed a dose reduction of dabrafenib to 50 mg once daily due to pyrexia. In the subsequent 10 patients, the trametinib dose was reduced in 5 patients (because of elevated liver enzymes and pneumonitis; central serous retinopathy; fever and elevated liver enzymes; decreased ejection fraction; and hyponatremia and syncope); the dabrafenib dose was reduced in 4 patients (relapsing pneumonitis; pyrexia and elevated liver enzymes; vomiting and diarrhea; and hyponatremia and syncope). There were no permanent treatment discontinuations due to AE.

### 3.4. Efficacy

The tumor response rate for the intent-to-treat population (*n* = 16 patients) was 1 partial response (duration 15.4 weeks), and 7 stable diseases (ORR 6.3%; disease control rate [DCR] 50.0%) (Figure 2, Table 3, Figures S1 and S2).

**Figure 3:**
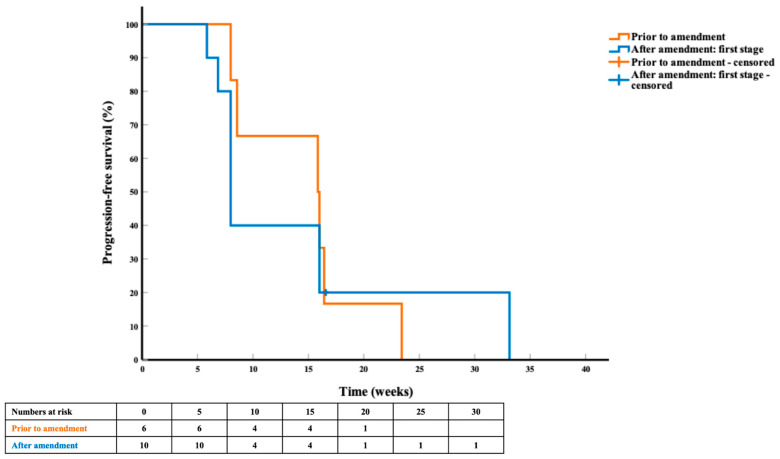
Progression-free survival curve and life table in the first six patients enrolled prior to trial amendment (orange) and in the ten patients enrolled after trial amendment in the first stage of the study (blue).

**Figure 4:**
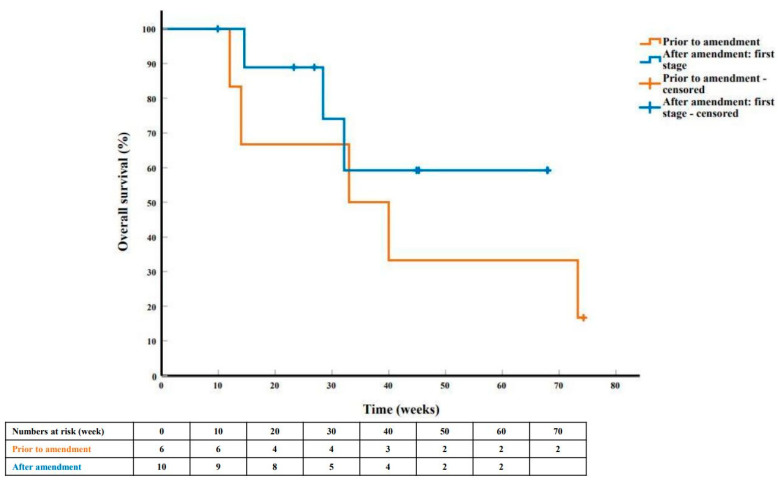
Overall survival curve and life table in the first six patients enrolled prior to trial amendment (orange) and in the ten patients enrolled after trial amendment in the first stage of the study (blue).

At the time of analysis, 8 patients (5 out of the first 6, and 3 out of the subsequent 10 patients) had died. The median duration of follow-up for surviving patients was 45.3 weeks (range 9.9–74.3). Fifteen patients (6 prior to and 9 after trial amendment) had progressed, the median PFS for the first 6 and subsequent 10 patients was 15.9 weeks (95% CI 6.9–24.8) and 8.0 weeks (95% CI 7.1–8.9), respectively (Figure 3). The median OS was 33.0 weeks (95% CI 1.8–64.2), and not reached (Figure 4), respectively. There was no significant difference in PFS and OS by *NRAS^Q61^* subtype mutation and by prior immunotherapy regimen.

### 3.5. Exploratory Endpoints

In the intent-to-treat population (*n* = 16 patients), the presence of detectable levels of *NRAS^Q61R/K/L^* mutant ctDNA in the plasma at baseline was associated with a trend towards a worse PFS (median PFS 8.0 [95% CI 6.7–9.3] versus 15.9 weeks [95% CI 4.3–27.4]; HR 3.324; *p =* 0.068) (Figure S3). A numerically but not statistically significantly improved OS was also observed (median OS 73.3 [95% CI not evaluable] versus 28.4 weeks [95% CI 7.3–49.5]; HR 0.655; *p =* 0.418) when baseline mutant ctDNA was absent (Figure S3).

Plasma *NRAS^Q61R/K/L^* mutant ctDNA was never detected in 5 patients during the course of the study (Table S2). In three additional patients with undetectable levels at baseline, ctDNA was detected before or at the timepoint of PD. Five patients with detectable levels of *NRAS^Q61R/K/L^* mutant ctDNA at baseline that persisted during therapy all experienced early progression of their disease.

A baseline TMTV >80 mL (assessed by ^18^F-FDG-PET/CT imaging in 15 patients) was associated with a trend towards worse OS (median OS 28.4 [95% CI 0.0–56.9] versus 73.3 weeks [95% CI not evaluable]; HR 3.161; *p* = 0.075) (Figure S4).

## 4. Discussion

This phase 2 clinical trial failed to meet its prespecified objective of documenting 2 or more confirmed objective responses among the first 10 ICI-pretreated patients with advanced *NRAS^Q61R/K/L^* mutant melanoma treated with trametinib and low-dose dabrafenib. In the first six patients enrolled prior to the trial amendment, one confirmed partial response was observed, while no responses were observed in the 10 patients who were enrolled after trial amendment. Disease control was achieved in 4 out of 6 and 4 out of 10 patients, respectively, suggesting that trametinib plus low-dose dabrafenib has some, albeit low and generally short-term activity in this population. Most patients in this study (14 of 16) had baseline stage IV-M1c or stage IV-M1d disease with extensive organ involvement (median of 5 affected organs), which might explain this observed low efficacy. Another possible explanation for the low efficacy of this combination is dabrafenib-induced paradoxical MAPK-pathway activation in *NRAS^Q61R/K/L^* mutant cancer cells. The use of BRAF-inhibitor monotherapy (e.g., dabrafenib) in advanced *BRAF^V600E/K^* mutant melanoma is associated with the development of secondary skin neoplasms (such as cutaneous squamous cell carcinomas and keratoacanthomas). Pathophysiologically, BRAF-inhibitors can bind to and activate wild-type RAF leading to “paradoxical” MAPK-pathway activation in *BRAF^V600^* wild-type skin cells, which manifests clinically as skin neoplasms [11]. Characterization of these neoplasms has shown a high prevalence of *NRAS* mutations that additionally drive the MAPK-pathway [12]. Combining full-dose BRAF-inhibition with downstream MEK-inhibition (e.g., dabrafenib plus trametinib) efficiently mitigates detrimental paradoxical activation of the MAPK-pathway, which significantly decreases the incidence of secondary neoplasms. However, the addition of the BRAF-inhibitor dabrafenib to a *NRAS^Q61R/K/L^* mutant cancer cell, as investigated in our trial, might have paradoxically activated downstream signaling through RAF in the MAPK-pathway, which was possibly insufficiently inhibited by the downstream MEK-inhibitor trametinib in a subset of patients without any clinical benefit. Finally, mutant *NRAS* does not only activate the MAPK-pathway, but can also transmit oncogenic signaling through the phosphoinositide-3-kinase pathway, as well as other pathways [13]. These downstream signaling pathways are not targeted by trametinib and low-dose dabrafenib.

All six patients enrolled prior to trial amendment developed symptomatic and disfiguring, treatment-limiting trametinib-related acneiform rash (5 grade 2; 1 grade 1). Al-though rash associated with MEK-inhibitor monotherapy may improve over time, the addition of low-dose dabrafenib to the same trametinib dosing after resolution of the rash to grade 0–1 prevented clinically relevant recurrences and enabled these patients to maintain maximal trametinib dosing.

Furthermore, the upfront addition of low-dose dabrafenib to a normal dose of trametinib, as has been investigated after amendment of the trial, primarily prevented the development of dose-limiting significant trametinib-related skin toxicity in all 10 patients (grade 1 acneiform rash in 30.0%). This is lower than the incidence of 57% with trametinib monotherapy in the phase 3 METRIC trial in advanced *BRAF^V600E/K^* mutant melanoma and of 65% with binimetinib monotherapy in the phase 3 NEMO trial in advanced *NRAS^Q61R/K/L^* mutant melanoma [5,7]. While we acknowledge that trametinib-related skin toxicity in the first 6 patients was low grade, we estimate that the CTCAE version 4.03 are not able to fully differentiate between the severity of acneiform rash of patients treated with trametinib monotherapy and with the combination.

Thus, these findings confirm earlier observations that the incidence of acneiform rash is lower with the combination of dabrafenib and trametinib compared to trametinib monotherapy. However, the novelty of this trial is that it provides convincing evidence that the cutaneous toxicity-limiting effect is already observed at low doses of dabrafenib (one third of the standard 150 mg twice daily dose that is used in *BRAF^V600E/K^* mutant melanoma). Low-dose dabrafenib did not increase the incidence of dabrafenib-related pyrexia in the majority of patients. Unfortunately, while successfully mitigating trametinib-related skin toxicity, the upfront addition of dabrafenib 50 mg twice daily was not able to achieve a higher dosing intensity of trametinib (90% versus 75%). Other trametinib- and dabrafenib-related toxicities occurred in both cohorts, at similar incidences to historical controls, and were managed according to available guidelines that were incorporated in the study protocol. The incidence of high-grade and serious AE, as well as 2 toxicities of special interest (drug-induced acute tubulointerstitial nephritis and interstitial pneumonitis) suggests the possibly lower tolerance of molecular-targeted therapies in patients who were previously treated with ICI, as has been suggested in previous research [14].

The association of presence of baseline *NRAS^Q61R/K/L^* mutant ctDNA with worse PFS with near-significance, as well as the association of high TMTV with worse OS with near-significance confirms earlier research by our group [10,15,16].

## 5. Conclusions

Trametinib plus low-dose dabrafenib is insufficiently active in previously treated advanced *NRAS^Q61R/K/L^* mutant melanoma patients. Addition of low-dose dabrafenib to trametinib significantly mitigates the risk of trametinib-related cutaneous toxicity, although upfront addition of low-dose dabrafenib does not allow reaching a higher dose intensity of trametinib. This newly defined dosing regimen may be of interest for further evaluation in patients with MEK-inhibitor-sensitive tumors [17,18].

## Figures and Tables

**Table 1:** Baseline characteristics of patients enrolled prior to and after trial amendment.

Baseline Characteristics	Patients Enrolled Prior to Trial Amendment *n* = 6	Patients Enrolled after Trial Amendment *n* = 10
Sex (*n* (%))		
Male	4 (66.7)	4 (40.0)
Female	2 (33.3)	6 (60.0)
Age (median (range))	70 (44–77)	58 (30–84)
ECOG PS (*n* (%))		
0	5 (83.3)	4 (40.0)
1	1 (16.7)	5 (50.0)
2	0	1 (10.0)
Melanoma subtype (*n* (%))		
Superficial spreading	2 (33.3)	5 (50.0)
Nodular	0	4 (40.0)
Lentigo maligna	1 (16.7)	0
Spitzoid	1 (16.7)	0
Cutaneous NOS	1 (16.7)	0
Unknown primary lesion	1 (16.7)	1 (10.0)
AJCC stage (*n* (%))		
IIIC	0	1 (10.0)
IV-M1b	0	1 (10.0)
IV-M1c	6 (100.0)	5 (50.0)
IV-M1d	0	3 (30.0)
Number of affected organs		
Median (range)	5 (2–7)	5 (1–8)
Lactate dehydrogenase (*n* (%))		
Increased	2 (33.3)	4 (40.0)
Normal	4 (66.7)	6 (60.0)
*NRAS* mutation subtype (*n* (%))		
*Q61R*	3 (50.0)	6 (60.0)
*Q61K*	3 (50.0)	3 (30.0)
*Q61L*	0	1 (10.0)
Prior lines of therapy		
Median (range)	2.5 (2–4)	2.5 (1–5)
1 (*n* (%))	0	3 (30.0)
2 (*n* (%))	3 (50.0)	5 (50.0)
3 (*n* (%))	2 (33.3)	0
>3 (*n* (%))	1 (16.7)	2 (20.0)
Prior PD-1 ICI (*n* (%))	6 (100.0)	9 (90.0)
Prior CTLA-4 ICI (*n* (%))	5 (83.3)	6 (60.0)
Prior PD-1 ICI/CTLA-4 ICI combination (*n* (%))	1 (16.7)	3 (30.0)
*NRAS^Q61R/K/L^* mutant ctDNA (*n* (%))		
Present	2 (33.3)	4 (40.0)
Absent	4 (66.7)	6 (60.0)
Total metabolic tumor volume mL (median (range))	123 (3–4392)	44 (0–614) *

* Only 9 patients underwent baseline whole-body ^18^F-FDG-PET/CT imaging. Abbreviations: AJCC: American Joint Committee on Cancer; ctDNA: circulating tumor DNA; CTLA-4 ICI: cytotoxic T-lymphocyte-associated antigen 4 immune checkpoint inhibitor; ECOG PS: Eastern Cooperative Oncology Group Performance Status; NOS: not otherwise specified; PD-1 ICI: programmed cell death 1 immune checkpoint inhibitor.

**Table 2:** Adverse events in patients enrolled prior to the trial amendment (*n* = 6) and in patients enrolled after the trial amendment (*n* = 10).

Adverse Events (*n* (%))	Patients Enrolled Prior to Trial Amendment *n* = 6		Patients Enrolled after Trial Amendment *n* = 10	
	All-Grade	Grade 3–4	All-Grade	Grade 3–4
All AE	6 (100.0)	3 (50.0)	10 (100.0)	4 (40.0)
Acneiform rash	6 (100.0) *	0	3 (30.0) °	0
Acneiform rash leading to temporary treatment interruption	6 (100.0)	0	0	0
Acneiform rash leading to add-on of low-dose dabrafenib	6 (100.0)	0	NA	NA
Fatigue	4 (66.7)	1 (16.7)	4 (40.0)	0
Creatine phosphokinase increase	4 (66.7)	0	6 (60.0)	0
Lipase increased	3 (50.0)	0	3 (30.0)	0
Anemia	3 (50.0)	0	2 (20.0)	0
Fever	3 (50.0)	0	1 (10.0)	1 (10.0)
Arterial hypertension	2 (33.3)	0	3 (30.0)	0
Aspartate aminotransferase increase	2 (33.3)	0	3 (30.0)	1 (10.0)
Central serous retinopathy	2 (33.3)	0	1 (10.0)	0
Diarrhea	2 (33.3)	0	4 (40.0)	0
Lymphocyte count decreased	2 (33.3)	0	1 (10.0)	0
Nausea	2 (33.3)	0	3 (30.0)	0
Hyperkalemia	1 (16.7)	1 (16.7)	0	0
Hyponatremia	1 (16.7)	1 (16.7)	3 (30.0)	2 (20.0)
Idiopathic thrombocytopenic purpura	1 (16.7)	1 (16.7)	0	0
Lung infection	1 (16.7)	1 (16.7)	0	0
Syncope	1 (16.7)	1 (16.7)	1 (10.0)	1 (10.0)
Abdominal pain	1 (16.7)	0	1 (10.0)	0
Alanine aminotransferase increase	1 (16.7)	0	3 (30.0)	2 (20.0)
Anorexia	1 (16.7)	0	2 (20.0)	0
Atrial fibrillation	1 (16.7)	0	0	0
Blot bleed retina	1 (16.7)	0	0	0
Chills	1 (16.7)	0	2 (20.0)	0
Cough	1 (16.7)	0	0	0
Edema lower limbs	1 (16.7)	0	1 (10.0)	0
Eosinophilia	1 (16.7)	0	0	0
Ejection fraction decreased	1 (16.7)	0	1 (10.0)	1 (10.0)
Eosinophil count increased	1 (16.7)	0	0	0
Heart failure	1 (16.7)	0	0	0
Hypoalbuminemia	1 (16.7)	0	0	0
Ileus	1 (16.7)	0	0	0
Inflammatory syndrome	1 (16.7)	0	0	0
Maculopapular rash	1 (16.7)	0	1 (10.0)	0
Malaise	1 (16.7)	0		
Neutrophil count decreased	1 (16.7)	0	3 (30.0)	0
Occlusion retinal arteriolus	1 (16.7)	0	0	0
Operculum retinae	1 (16.7)	0	0	0
Paronychia	1 (16.7)	0	0	0
Platelet count decreased	1 (16.7)	0	2 (20.0)	0
Radiation pneumonitis	1 (16.7)	0	0	0
White blood cell count decreased	1 (16.7)	0	2 (20.0)	1 (10.0)
Vomiting	0	0	3 (30.0)	0
Acute kidney injury	0	0	2 (20.0)	0
Muscle cramps	0	0	2 (20.0)	0
Gamma glutamyl transferase increased	0	0	1 (10.0)	1 (10.0)
Pneumonitis	0	0	1 (10.0)	1 (10.0)
Pulmonary embolism	0	0	1 (10.0)	1 (10.0)
Alkaline phosphatase increased	0	0	1 (10.0)	0
Arthralgia	0	0	1 (10.0)	0
Bronchopulmonary hemorrhage	0	0	1 (10.0)	0
Dry mouth	0	0	1 (10.0)	0
Dyspepsia	0	0	1 (10.0)	0
Headache	0	0	1 (10.0)	0
Hypokalemia	0	0	1 (10.0)	0
Neuropathic pain	0	0	1 (10.0)	0
Posterior vitreous detachment	0	0	1 (10.0)	0
Retinal pigment epithelial detachment	0	0	1 (10.0)	0
Retinoschisis	0	0	1 (10.0)	0
Squamous rash	0	0	1 (10.0)	0
Skin infection	0	0	1 (10.0)	0
Urinary tract infection	0	0	1 (10.0)	0
Serious AE	3 (50.0)	2 (33.3)	5 (50.0)	4 (40.0)
AE leading to dose reduction	3 (50.0)	0	5 (50.0)	4 (40.0)
AE leading to temporary treatment interruption	6 (100.0)	2 (33.3)	6 (60.0)	4 (40.0)
AE leading to permanent treatment interruption	0	0	0	0

* Five patients with grade 2 and one patient with grade 1; ° three patients with grade 1. Abbreviations: AE: adverse events; NA: not applicable.

**Table 3:** Best objective response in patients enrolled prior to the trial amendment, and in patients enrolled after the trial amendment and who were part of the first stage of the study.

Best Objective Response (*n* (%))	Patients Enrolled Prior to Trial Amendment *n* = 6	Patients Enrolled after Trial Amendment *n* = 10
Confirmed objective response	1 (16.7) *	0
Complete response	0	0
Partial response	1 (16.7) *	0
Stable disease	3 (50.0) °	4 (40.0)
Progressive disease	2 (33.3)	6 (60.0)
Objective response rate	1 (6.3)	
Disease control rate	8 (50.0)	

* Duration of response 15.4 weeks; ° 2 unconfirmed partial responses.

## Data Availability

The data presented in this study are available upon reasonable request from the corresponding author. The data are not publicly available due to ethical/privacy reasons.

## Supplementary Materials

The following are available online at https://www.mdpi.com/article/10.3390/cancers13092010/s1, Table S1: Inclusion and exclusion criteria, Table S2: Evolution of *NRAS^Q61R/K/L^* mutant ctDNA at baseline, during therapy and at progression of disease, Figure S1: Swimmer plot depicting the change in sum of diameters of target lesions in the patients enrolled prior to trial amendment (orange) (*n* = 6) and patients enrolled after trial amendment in the first stage of the study (blue) (*n* = 8). One patient enrolled after the amendment was evaluated by clinical assessment (in-transit lesions), another patient was not evaluable due to early progressive disease. Circle: new lesions only; square: progression of non-target lesions only; triangle: new lesions and progression of non-target lesions. Abbreviations: SDTL: sum of diameters of target lesions, Figure S2: Waterfall plot depicting the maximal change in sum of diameters of target lesions in the patients enrolled prior to trial amendment (orange) (*n* = 6) and patients enrolled after trial amendment in the first stage of the study (blue) (*n* = 8). One patient enrolled after the amendment was evaluated by clinical assessment (in-transit lesions), another patient was not evaluable due to early progressive disease. Abbreviations: SDTL: sum of diameters of target lesions, Figure S3: (a) Progression-free survival in all study patients (*n* = 16) (prior to and after trial amendment) by baseline *NRAS^Q61R/K/L^* mutant ctDNA presence; (b) overall survival in all study patients (*n* = 16) (prior to and after trial amendment) by baseline *NRAS^Q61R/K/L^* mutant ctDNA presence. Abbreviations: ctDNA: circulating tumor DNA, Figure S4: Overall survival curve in all study patients (*n* = 16) (prior to and after trial amendment) by baseline TMTV on ^18^F-FDG PET/CT. Abbreviations: ^18^F-FDG PET/CT: 18-fluorodeoxyglucose positron emission tomography/computed tomography; TMTV: total metabolic tumor volume.
