# Supplementary material for: A Phase 2 Clinical Trial of Trametinib and Low-Dose Dabrafenib in Patients with Advanced Pretreated NRASQ61R/K/L Mutant Melanoma (TraMel-WT)
Source: Cancers (Basel). 2021 Apr 22;13(9):2010. doi: 10.3390/cancers13092010 (PMC8122428; doi:10.3390/cancers13092010)
Supplement: Supplementary file 1 [file cancers-13-02010-s001.zip › cancers-1136061-supplementary.pdf]

cancers

MDPI

Article

Supplementary Materials: A Phase 2 Clinical Trial of Trametinib and Low-Dose Dabrafenib in Patients with Advanced Pretreated *NRAS*$^{Q61R/K/L}$ Mutant Melanoma (TraMel-WT)

Gil Awada, Julia Katharina Schwarze, Jens Tijtgat, Giuseppe Fasolino, Hendrik Everaert and Bart Neyns

Table S1. Inclusion and exclusion criteria.

Inclusion criteria

Subjects eligible for enrolment in the study must meet all of the following criteria:

- ≥ 18 years of age;
- Signed written informed consent;
- Histologically confirmed advanced melanoma that is either stage III (unresectable) or stage IV (metastatic);
- Absence of a *BRAF*^{V600} mutation as determined by a validated test;
- In case of mucosal or acral melanoma, absence of a *cKIT* mutation as determined by a validated test;
- Presence of archival melanoma tissue of possibility of new biopsy for mutational testing;
- Subjects must have failed at least one prior systemic treatment with immune checkpoint inhibitors: CTLA-4 blocking immune checkpoint inhibitors (ipilimumab or other experimental anti-CTLA-4 antibodies), PD-1 blocking immune checkpoint inhibitors (pembrolizumab, nivolumab or other experimental anti-PD-1 antibodies) and/or PD-L1 blocking immune checkpoint inhibitors (avelumab, atezolizumab, durvalumab or other experimental anti-PD-L1 antibodies). Progression of disease per RECIST, version 1.1, or per immune related response criteria (irRC) must have been documented during this treatment. Patients who are not able to undergo such treatment are also eligible.
- The presence of at least one measurable lesion per RECIST, version 1.1
- Interval between the date of the last administration of prior therapy for melanoma and the date of recruitment:
    - ≥ 12 weeks following the date of the first administration and ≥4 weeks following the date of the last administration of CTLA-4, PD-1 or PD-L1 blocking immune checkpoint inhibitor;
    - ≥ 4 weeks following the date of the last administration of chemotherapy (≥ 6 weeks in case of a nitrosurea or mitomycin C containing regimen);
    - ≥ 4 weeks following major surgery or extensive radiotherapy.
- All prior anti-cancer treatment-related toxicities (except alopecia and laboratory values as listed on Table 1 must be ≤grade 1 according to the Common Terminology Criteria for Adverse Events version 4 (CTCAE version 4.03; National Cancer Institute [NCI] 2010) at the time of recruitment.
- Able to swallow and retain oral medication and must not have any clinically significant gastrointestinal abnormalities that may alter absorption such as malabsorption syndrome or major resection of the stomach or bowels.
- Women of childbearing potential must have a negative serum pregnancy test within 14 days prior to recruitment and agree to use effective contraception throughout the treatment period, and for 16 weeks after the last dose of study treatment.
- Men with a female partner of childbearing potential must have either had a prior vasectomy or agree to use effective contraception from 14 days prior to administration of the first dose of study

treatment, throughout the treatment period, and for 16 weeks after the last dose of study treatment.
- An Eastern Cooperative Oncology Group (ECOG) performance status of 0, 1 or 2.
- Adequate baseline organ function as defined in Table 1.

Table 1. Definitions of adequate baseline organ function.

System	Laboratory values
Hematologic	
Absolute neutrophil count	≥ 1.2 x 10^3/mm^3
Hemoglobin	≥ 9.0 g/dL
Platelet count	≥ 75 x 10^3/mm^3
PT/INR and APTT[a]	≤ 1.5 x ULN
Hepatic	
Albumin	≥ 2.5 g/dL
Total bilirubin	≤ 1.5 x ULN
AST and ALT	≤ 2.5 x ULN
Renal	
Calculated creatinine clearance[b]	≥ 50 mL/min
Cardiac	
Left ventricular ejection fraction	≥ LLN by transthoracic echocardiogram

Abbreviations: APTT: activated partial thromboplastin time; ALT: alanine aminotransferase; AST: aspartate aminotransferase; INR: international normalized ratio; LLN: lower limit of normal; PT: prothrombin time; ULN: upper limit of normal.
a. Increase is allowed in the context of anticoagulant use.
b. By use of the Cockroft-Gault formula.

Exclusion criteria

Subjects meeting any of the following criteria must not be enrolled in the study:
- Subjects with uveal melanoma.
- Prior treatment with MAPK-pathway inhibitors (BRAF inhibitors, MEK inhibitors)
- Subjects with clinically active brain metastases (lesions should be stable and have been definitely treated with stereotactic radiation therapy, surgery or gamma knife therapy with no evidence of disease progression prior to enrollment.
- Any contra-indication for evaluation by whole body FDG-PET/CT and MRI of the brain.
- History of another malignancy. Exception: subjects who have been disease-free for 3 years, (i.e. subjects with second malignancies that are indolent or definitively treated at least 3 years ago) or subjects with a history of completely resected non-melanoma skin cancer.
- Current use of any prohibited medication.
- Taken an investigational drug within 28 days or 5 half-lives (minimum 14 days), whichever is shorter, prior to recruitment.
- Any serious or unstable pre-existing medical conditions (aside from malignancy exceptions specified above), psychiatric disorders, or other conditions that could interfere with the subject's safety, obtaining informed consent, or compliance with study procedures.
- Known Human Immunodeficiency Virus (HIV), hepatitis B virus (HBV), or hepatitis C virus (HCV) infection (subjects with laboratory evidence of cleared HBV and HCV infection will be permitted).
- No enzyme inducing anticonvulsants for ≥4 weeks prior to recruitment
- A history or evidence of cardiovascular risk including any of the following:
    - Current LVEF < LLN;
    - A QT interval corrected (QTc) for heart rate using the Bazett's formula (QTcB) ≥ 480 milliseconds;
    - A history or evidence of current clinically significant uncontrolled arrhythmias. Exception: subjects with atrial fibrillation controlled for > 30 days prior to recruitment are eligible.

- A history (within 6 months prior to recruitment) of acute coronary syndromes (including myocardial infarction or unstable angina), or coronary angioplasty;
- A history or evidence of current ≥ Class II congestive heart failure as defined by the New York Heart Association (NYHA) guidelines;
- Treatment refractory hypertension defined as a blood pressure of systolic >140 mmHg and/or diastolic > 90 mm Hg which cannot be controlled by antihypertensive therapy;
- Patients with intra-cardiac defibrillators or permanent pacemakers;
- Known cardiac metastases;
- Abnormal cardiac valve morphology (≥ grade 2) documented by echocardiogram (subjects with grade 1 abnormalities [i.e., mild regurgitation/stenosis] can be entered on study). Subjects with moderate valvular thickening should not be entered on study.
- Uncorrectable electrolyte abnormalities (e.g. hypokalemia, hypomagnesemia, hypocalcemia), long QT syndrome or taking medicinal products known to prolong the QT interval.

- A history or current evidence/risk of retinal vein occlusion (RVO) or central serous retinopathy (CSR) including:
    - Presence of predisposing factors to RVO or CSR (e.g., uncontrolled glaucoma or ocular hypertension, uncontrolled hypertension, uncontrolled diabetes mellitus, or a history of hyperviscosity or hypercoagulability syndromes); or
    - Visible retinal pathology as assessed by ophthalmic examination that is considered a risk factor for RVO or CSR such as:
    - Evidence of new optic disc cupping;
    - Evidence of new visual field defects on automated perimetry;
    - Intraocular pressure >21 mmHg as measured by tonography.
- Known immediate or delayed hypersensitivity reaction or idiosyncrasy to drugs chemically related to the study treatments, their excipients, and/or dimethyl sulfoxide (DMSO).
- Females who are nursing.

Table S2. Evolution of *NRAS*$^{Q61R/K/L}$ mutant ctDNA at baseline, during therapy and at progression of disease.

**Patient Number**	**ctDNA**			**Efficacy**				
**Prior to Trial Amendment**	**Baseline**	**On Therapy**	**PD**	**BOR**	**PD**	**PFS (weeks)**	**Dead**	**OS (weeks)**
10	+	+	- *	SD	Yes	16.0	No	74.3
8	-	-	+	SD	Yes	15.9	Yes	73.3
6	-	-	-	PR	Yes	23.4	Yes	40.0
1	-	-	?°	SD	Yes	16.4	Yes	33.0
3	-	+	+	PD	Yes	8.6	Yes	14.0
4	+	+	+	PD	Yes	8.0	Yes	12.0
**After Trial Amendment**	**Baseline**	**During Therapy**	**PD**	**BOR**	**PD**	**PFS (weeks)**	**Dead**	**OS (weeks)**
12	-	-	-	SD	Yes	33.1	No	68.0
11	-	-	-	SD	Yes	16.0	No	67.9
18	+	-	-	PD	Yes	8.0	No	45.3
19	-	+	+	PD	Yes	8.0	No	44.9
16	+	+	+	PD	Yes	6.9	Yes	32.1
17	+	+	+	SD	Yes	16.0	Yes	28.4
22	-	-	-	PD	Yes	8.0	No	26.9
23	-	-	NA	SD	No	16.6	No	23.3
14	+	+	+	PD	Yes	5.9	Yes	14.6
26	-	-	-	PD	Yes	8.0	No	9.9

* Patient 10 had presence of ctDNA at the timepoint before and after the moment of PD. ° Patient 1 did not undergo sampling for ctDNA at the moment of progression. Abbreviations: BOR: best objective response; ctDNA: circulating tumor DNA; NA: not applicable; OS: overall survival; PD: progressive disease; PFS: progression-free survival; PR: partial response; SD: stable disease.

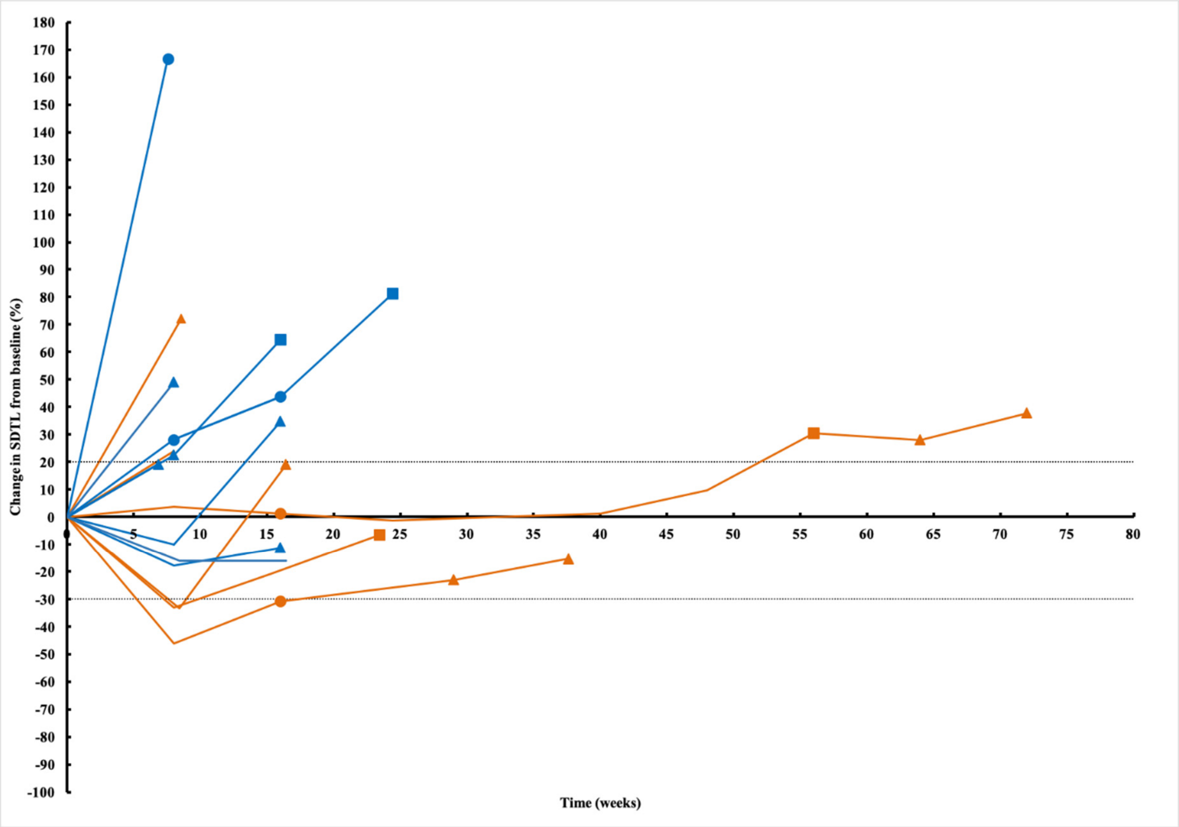

Figure S1. Swimmer plot depicting the change in sum of diameters of target lesions in the patients enrolled prior to trial amendment (orange) (n = 6) and patients enrolled after trial amendment (blue). One patient enrolled after the amendment was evaluated by clinical assessment (in-transit lesions), another patient was not evaluable due to early progressive disease (n = 8). Circle: new lesions only; square: progression of non-target lesions only; triangle: new lesions and progression of non-target lesions. Abbreviations: SDTL: sum of diameters of target lesions.

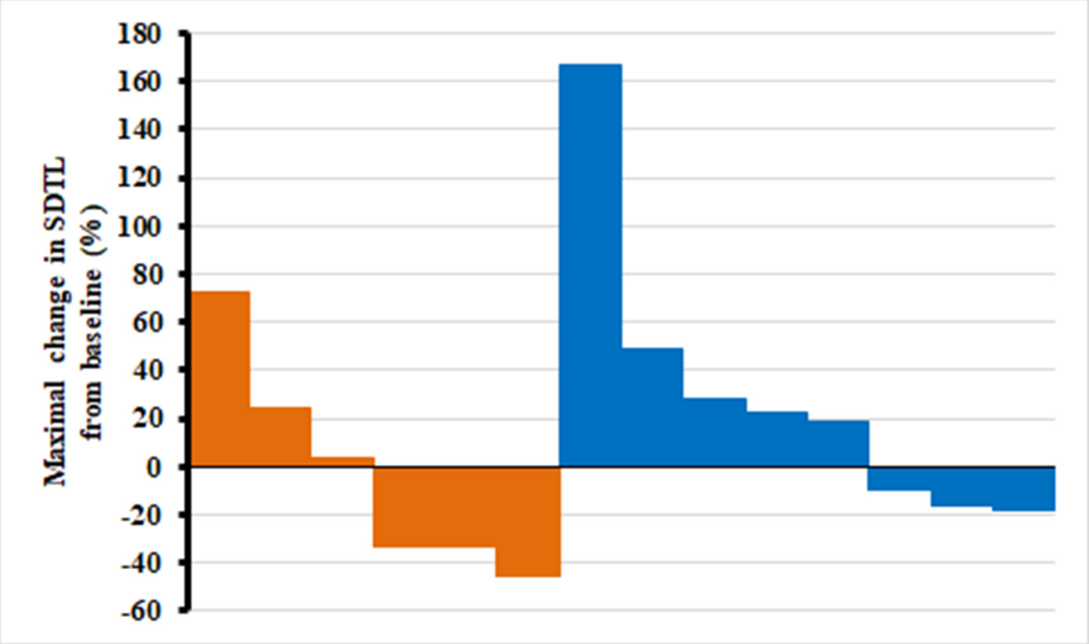

Figure S2. Waterfall plot depicting the maximal change in sum of diameters of target lesions in the patients enrolled prior to trial amendment (orange) (n = 6) and patients enrolled after trial amendment (blue) (n = 8). One patient enrolled after the amendment was evaluated by clinical assessment (in-transit lesions), another patient was not evaluable due to early progressive disease. Abbreviations: SDTL: sum of diameters of target lesions.

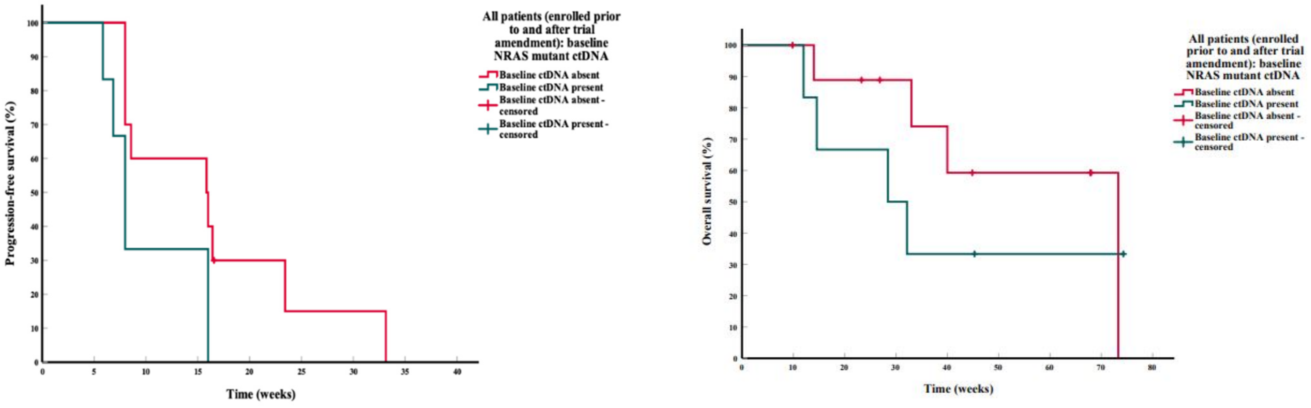

(a) (b)

Figure S3. (a) Progression-free survival in all study patients (*n* = 16) (prior to and after trial amendment) by baseline *NRAS*$^{Q61R/K/L}$ mutant ctDNA presence; (b) overall survival in all study patients (*n* = 16) (prior to and after trial amendment) by baseline *NRAS*$^{Q61R/K/L}$ mutant ctDNA presence. Abbreviations: ctDNA: circulating tumor DNA.

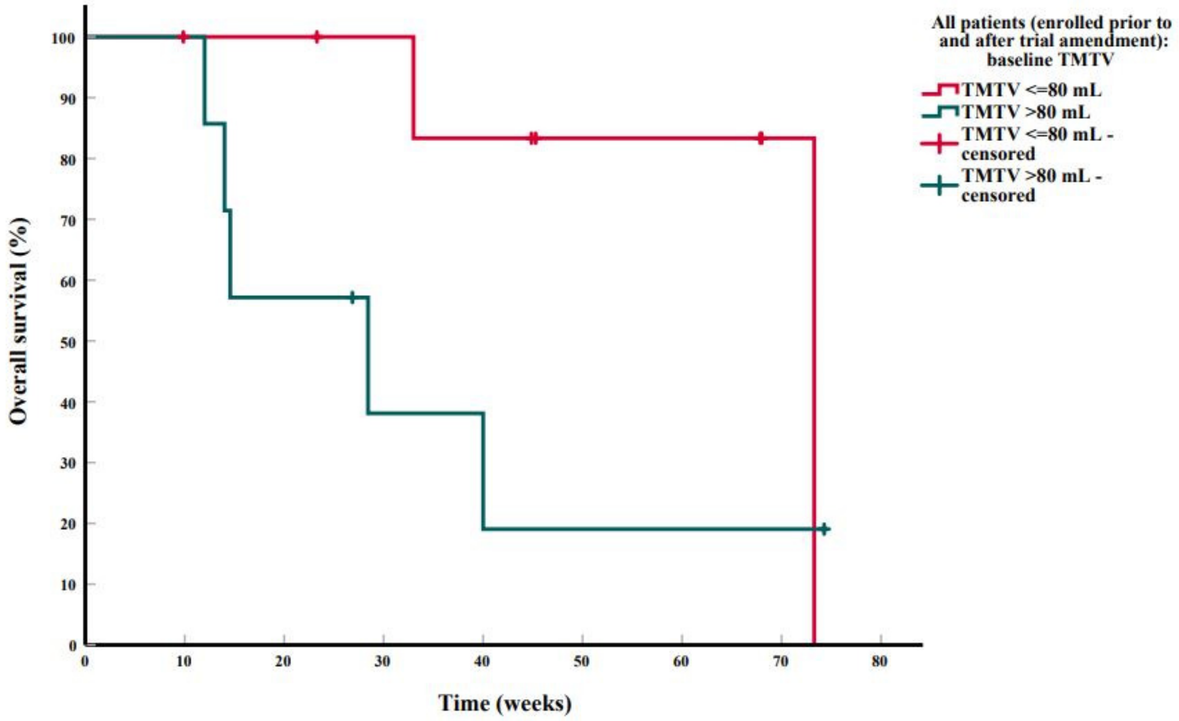

Figure S4. Overall survival curve in all study patients (*n* = 16) (prior to and after trial amendment) by baseline TMTV on ^{18}F-FDG PET/CT. Abbreviations: ^{18}F-FDG PET/CT: 18-fluorodeoxyglucose positron emission tomography/computed tomography; TMTV: total metabolic tumor volume.
